# Evaluation of the Wound Healing Activity of a Traditional Compound Herbal Product Using Rat Excision Wound Model

**Published:** 2017

**Authors:** Maryam Jahandideh, Homa Hajimehdipoor, Seyed Alireza Mortazavi, Ahmadreza Dehpour, Gholamreza Hassanzadeh

**Affiliations:** a *Department of Pharmacognosy, Faculty of Pharmacy, Pharmaceutical Sciences Branch, Islamic Azad University, Tehran, Iran (IAUPS). *; b *Traditional Medicine and Materia Medica Research Center and Department of Traditional Pharmacy, School of Traditional Medicine, Shahid Beheshti University of Medical Sciences, Tehran, Iran.*; c *Department of Pharmaceutics, * *School * *of * *Pharmacy, Shahid Beheshti University * *of * *Medical Sciences, * *Tehran, Iran. *; d *Department of Pharmacology, School of Medicine, Tehran University of Medical Sciences, Tehran, Iran.*; e *Experimental Medicine Research Center ,Tehran University of Medical Sciences ,Tehran ,Iran.*; f *Department of Anatomy, School of Medicine, Tehran University of Medical Sciences, Tehran, Iran.*

**Keywords:** *Boswellia carteri*, *Aloe vera*, *Commiphora myrrha*, wound healing, excision wound model, Iranian Traditional Medicine

## Abstract

Iranian Traditional Medicine (ITM) manuscripts contain prescriptions that have long been used for healing of wounds. The present study evaluates the healing effect of a poly herbal paste (PHP), retrieved from ITM sources, containing *Aloe vera, Commiphora myrrha *and* Boswellia carteri* using rat excision wound model.

Excision wounds were induced in six groups consisting of six rats each. Group 1 received no treatment, while groups 2 and 6 received tetracycline ointment, Alpha ointment, PHP 40%, PHP 10% and paste base every day, respectively. The percentage of wound contraction on days 2, 7, 14 and 21 and histopathology parameters of healed wounds on 14^th^ and 21^st^ days were evaluated. Moreover, antioxidant activity of PHP was evaluated using DPPH method.

There was a significant improvement in wound healing in PHP 10% group on the 7^th^ day of the treatment (p<0.05). Moreover, the healing effect of PHP 10% was significantly greater than the control, tetracycline and paste base groups on the 2^nd^, 14^th^ and 21^st^ days (p<0.05). On day 14, PHP 40% showed significant healing effect compared to the control, tetracycline and paste base groups (p<0.05). Fewer inflammatory cells were observed in PHP 10%-treated animals and this group demonstrated better re-epithelialization with remarkable neovascularization. Besides, the PHP 10% formulation exhibited antioxidant activity.

*In vivo* and histopathologic examinations showed considerable wound healing in PHP 10% group. This finding could probably be due to the antioxidant, anti-inflammatory and antimicrobial activities of phytoconstituents of *A. vera, B. carteri* and *C. myrrha*.

## Introduction

 Nowadays, traditional herbal medicine has been increasingly considered despite remarkable improvement in modern medicine. Plants, with a valuable traditional support, have been noted as potential agents for prevention and treatment of disorders in recent years, which have given rise to some important modern drugs. However, our knowledge about their medicinal and toxicological properties is still needed to be improved(1).

The present study focuses on the medicinal plants that have been listed repeatedly in Iranian Traditional Medicine (ITM) sources for their wound healing properties. In addition, the selected plants have been evaluated using rat excision wound model. 

Each year, millions of people experience burns, suffer from chronic wounds, or have acute wounds that become complicated by infection, dehiscence or problematic scarring (2). Wound healing is a process that can be divided into three different phases: inflammation, proliferation and maturation. It should be born in mind, however, that wound healing is not always a linear process; it can progress and regress through the phases in response to various intrinsic and extrinsic factors. If the wound healing process is affected negatively, it can result in chronic wounds. Therefore, local management of the wound is essential for non-delayed wound healing and prevention from the development of chronic wounds (3). Management of infection and inflammation are the keys to a successful wound healing. Wound infection is one of the important factors that delay healing (4). Some plants with antibacterial and anti-inflammatory properties have shown to be effective for wound healing (5, 6). 

Iranian Traditional Medicine (ITM) is a medical school which is rich with intact information about plants that have been used for generations to treat skin disorders, among many others. A review of ITM sources shows that herbal therapy was the major treatment prescribed by practitioners and scholars of Iranian Traditional Medicine for wound healing. The herbal drugs listed in ITM references as useful in treatment of wounds have healing properties, and are flesh growing and cicatrizant (7-9). In one repeated prescription, a combination of *Aloe vera *(L.) Burm.f. (aloe), *Commiphora myrrha *(Nees) Engl. (myrrh) and* Boswellia carteri *Birdw. (frankincense) is named as efficient for wound healing (7-9).

Boswellic acids are the main active components of *Boswellia carteri *(Burseraceae) resin. Boswellia has had ethnomedicinal use and its anti-inflammatory effects have been proven and have made it a candidate for treating rheumatoid arthritis and other inflammatory diseases (10). Moreover, oil of *Boswellia carteri* has shown antimicrobial activity (11). Anti-inflammatory, antinociceptive and antioxidant effects of other *Boswellia *species have been established as well (12).

Myrrh is a yellow-brown aromatic oleo gum resin obtained from the stems of a number of plants of the genus *Commiphora*, particularly *C. myrrha *(Burseraceae). It has been demonstrated that myrrh has a broad spectrum of biological properties including antibacterial, antinociceptive, anti-inflammatory and antiulcer activities. Moreover, it has been reported that myrrh has antioxidant and immunopotentiating properties (13). The resin of *Commiphora *species has long been used for treatment of mouth ulcers, wounds, fractures, inflammatory diseases and pain. Terpenoids, especially sesqui- and triterpenoids, are the most abundant constituents in this genus (14-16)

*Aloe vera* is a tropical or subtropical plant with turgid lace-shaped green leaves. The plant has been widely cultivated in China and used as a traditional medicine for wound healing, and as an anti-cancer and anti-viral agent. Several studies have shown aloe gel (derived from *A. vera*) to accelerate wound healing after systemic or topical administration. Several mechanisms have been proposed for the wound healing effects of *A. vera*; these include keeping the wound moist, increased epithelial cell migration, more rapid maturation of collagen and reduction in inflammation (17). The mucilaginous polysaccharides present in the clear pulp of *A. vera* leaf have been demonstrated to be the major responsible ingredient for its healing properties. Several polysaccharides have been detected or isolated from *A. vera* gel, including mannan, galactan, glucomannan, arabinorhamnogalactan, pectic substance and glucuronic acid-containing polysaccharide (18). *A. vera* polysaccharides have demonstrated antioxidant properties. In addition, *A. vera* has antioxidant, laxative, anti-microbial, anti-inflammatory, anti-cancer and anti-malaria effects (19) .

In Iranian Traditional Medicine (ITM), *B. carteri* has been used for wound healing in combination with *A. vera* and myrrh. According to ITM references, *Kondor* (*Boswellia carteri,* common name*: *frankincense) is a wound cleanser (*monaqqīī*); it is desiccative (*mojaffif*), detergent (*jālī*), hemostatic (*ḥābis od-dam*), styptic (*qābiḍ*) and cicatrizant (*modammil*), especially in the case of fresh wounds. Also, ITM references have registered that *Sabr-e zard* (*Aloe vera, *common name: aloe) is a desiccative agent and has wound healing activity. *Morr-e makki *(*Commiphora myrrha, *common name: myrrh*) *is detergent, desiccative, styptic, wound cleanser and wound healer (7-9).

In the present study, wound healing activity of a formulation containing equal amounts of *A. vera*, *C. myrrha* and *B. carteri* in rat wound model has been evaluated.

## Experimental

*Data extraction from ITM textbooks*

Sieving through the plants that have been used for wound healing according to ITM references demonstrated that the three plants: *A. vera*, *C. myrrha* and *B. carteri* were the most repeated and emphasized on. We extracted triple combinations of the mentioned plants with a ratio of 1:1:1 in a single prescription; the chosen combination was studied for more elaborate formulation (7-9, 20, 21).

*Plant materials*

*Aloe vera *(L.) Burm.f. (traditional name: *Sabr-e zard*, common name: Aloe) leaves were purchased from Institute of Medicinal Plants in Karaj. *Boswellia carteri* Birdw (traditional name: *Kondor* and common name: frankincense) and *Commiphora myrrha* (Nees) Engl. (traditional name: *Morr-e makki*, common name: myrrh) were purchased from the conventional herbal market of Tehran. The samples were authenticated by Mohammad Kamalinejad, Department of Pharmacognosy, School of Pharmacy, Shahid Beheshti University of Medical Sciences, Tehran, Iran. All voucher specimens were deposited at the herbarium of Traditional Medicine and Materia Medica Research Center (TMRC), Shahid Beheshti University of Medical Sciences, Tehran, Iran for future reference. 

*Chemicals*

2,2-diphenyl-1-picrylhydrazyl (DPPH) was prepared from Sigma-Aldrich, UK. All reagents and solvents were of analytical grade or of pure quality; all were purchased from Merck Company (Germany). Tetracycline and Alpha ointment (a herbal product prepared from *Lowsonia inermis*, well known as a conventional wound healing product in Iran) were purchased from Iran Darou and Pars Darou Companies (Iran), respectively.

*Preparation of herbal powders*

The oleo gum resin of *C. myrrha* and *B. carteri* were rinsed with water and dried at room temperature, after which they were powdered and passed through 40 mesh sieve.

Fresh *A. vera* leaves were sliced and the gel was separated from the leaves. Then the gel was freeze-dried. Temperature of the condenser and average chamber pressure were adjusted at -40 °C and 50 mL Tour (VirTis, benchtopSlC). After four days, aloe powder was obtained from frozen *A. vera* gel and was passed through 40 mesh sieve (22).

*Formulation of a topical preparation*

Based on the information extracted from ITM manuscripts, a herbal wound healing paste was prepared by integrating powders of *Aloe vera, Commiphora myrrha, Boswellia carteri *into a hydrophilic base. 

In order to prepare the hydrophilic base of the paste, carbomer 940 was dissolved in warm water (2%). Then NaOH 0.1 M solution was gradually added to the mixture until the gel was formed. The pH was also measured to achieve the desired pH.

Finally, the powdered mixture of three herbal materials (1:1:1) was added to the base. Two formulations were prepared: a) formulation containing 10% active ingredients, b) formulation containing 40% active ingredients. The maximum concentration of herbal powders, which was stable in the gel base, was 40%. Moreover, methyl and propyl parabens and sodium meta bisulfide were added to the product as microbial preservatives and antioxidant, respectively.

*Pharmacological study*

*Animals*

In this experiment, male Wistar rats weighting 200-250g were used. The rats were kept under controlled conditions of light (12 h light–dark cycles) and room temperature (23±1˚C). This study was undertaken after obtaining the approval of Ethics Committee of Shahid Beheshti University of Medical Sciences, no. 121.

*Wound* *induction (excision wound model)*

Rats were anaesthetized using an intra-peritoneal injection of ketamine 90 mg/kg (ketamine 10%, Alfasen, Woerden, Holland) with xylazine 10 mg/kg (xylazine 2%, Alfasen, Woerden, Holland). Then, the dorsal skin of the rats was depilated, and after disinfection of skin with Hexasept solution, full thickness round wounds (20 mm in diameter) were excised under aseptic conditions with the help of sterile dermal biopsy punch (23). Full thickness wounds were excised from the back of the rats using surgical scissors to the depth of loose subcutaneous tissues (24). Animals were divided into six groups (6 rats per group):

 Group 1: control, induced wound without treatment; Group 2: tetracycline ointment; Group3: Alpha ointment; Group 4: poly herbal paste 40% (PHP40%); Group 5: poly herbal paste 10 % (PHP10%); Group 6: paste base (poly herbal paste without active ingredients).

*Wound healing assessment*

*Rate of wound healing*

The rate of wound contraction was measured as the percentage of reduction from the original wound size every day, by taking picture with a digital camera. The pictures were taken from an equal distance from the wound and at a right angle to its surface. Before taking the picture, the wounds were disinfected by Hexasept solution to clean the wound surface and remove any debris. The wounds were bandaged again after taking the pictures. The captured images were examined by Image Mixle software to measure the wound size. The percentage of wound contraction was calculated using the following equation (25):

Wound contraction (%) = 100 × [(first day wound size – specific day wound size)/first day wound size]

*Histopathology*

On the 14^th^ and 21^st^ days, skin tissue samples from the wound and its vicinity were taken for histopathological study. Moreover, on the 14^th^ and 21^st^ days, kidney samples were taken for assessment of renal toxicity. Tissues were fixed in formalin 10% and embedded in paraffin. Sagittal sections (5μm thick) were prepared and stained with hematoxylin-eosin and photographed under 200 or 400× magnification by Optika light microscope and its morphometric software. In each sample, fibroblasts, macrophages, neutrophils and blood vessels were studied. Also, Optika software was used for capturing images of slides and measuring diameter of kidney lobules (26).

*2,2-Diphenyl-1-picrylhydrazyl (DPPH) radical scavenging assay*

DPPH radical scavenging assay is one of the most extensively used methods, which provides an easy and rapid way to evaluate the antiradical activities of herbal antioxidants. According to this colorimetric method, the antioxidant potential of a plant sample is associated with its scavenging activity of DPPH free radicals resulting in decolorization of the radical solution (27, 28). In the present study, to determine DPPH radical scavenging activity of PHP, methanol fraction of the paste was used (1:5 w/v). In brief, 100 µL of DPPH methanol solution (0.004% w/v) was added to 100 µL of serial dilutions (0.2-125mg/mL) of PHP methanol fraction in a 96-well micro-plate. After shaking for 30 min, the absorbance of the solutions was measured at 517 nm. During the experiment, all solutions were kept in darkness at room temperature. Mixture of 100 µL methanol with 100 µL PHP methanol fraction was used as the blank, while the negative control consisted of 100 µL DPPH solution plus 100 µL methanol. Butylated hydroxyltoluene (BHT) was used as positive control. Antioxidant activity was calculated using the following equation:

Scavenging capacity % = 100- [(A_S_ - A_B_) × 100/A_C_]

In which, A_S_, A_B_ and A_C_ are the absorbance of the sample, blank and the negative control, respectively. The concentration of PHC methanol fraction providing 50% inhibition (IC_50_) was calculated from the plot of inhibition percentage against PHC methanol fraction concentration. The tests were performed in triplicate.

*Statistical analysis*

All values were registered as mean ± S.D. Data were analyzed using one-way ANOVA, followed by Tukey's post hoc test. The results were considered significantly different at *p*<0.05.

## Results

*Rate of wound healing*

The percentage of wound healing for each group has been presented in table 1. Analysis of the data showed that the percentage of wound healing of PHP 10%-treated rats was significantly greater than control and tetracycline and paste base on the 2^nd^ day (*p*<0.05). The percentage of wound healing in PHP 10% -treated rats demonstrated significant difference with other groups on the 7^th ^day (*p*<0.050). As is observed in table 1, on the 14^th ^day, there was a significant difference between wound healing effects of PHP (10% and 40%) and control, tetracycline and paste base groups; but no difference was evident between PHP 10% and PHP 40% and Alpha ointment. On the 21^st^ day, the percentage of wound healing of PHP (10% and 40%) and Alpha -treated rats was significantly greater than control, but there was no difference between the three mentioned products.

**Table1:** Percentile reduction of wound size in control and treated rats

**Wound contraction (%)**				
**Group **	**2** ^nd^ ** day **	**7** ^th^ ** day**	**14** ^th^ ** day**	**21** ^st^ ** day**
control	-26.3 ±3.5	-2.7 ± 0.1	16.8 ± 4.2	55.0 ±5.0
tetracycline	-28.8± 4.7	-6.1 ±1.0	31.0 ±5.4	63.4 ±3.9
alpha	-13.6± 4.1	-2.1 ± 0.1	54.7 ±6.4*	80.6 ±3.8*
PHP 40%	-15.6± 3.4	-4.7 ±1.5	58.5 ±6.3*^a^^c^	78.1 ±3.2*
PHP 10%	-11.3± 2.1 *^a^^c^	16.2 ±3.2*^a^^b^^c^^d^	62.6 ±3.5*^a^^c^	84.1 ±2.5*^a^^c^
paste base	-27.3± 3.7	-4.2 ±1.3	19.1 ±4.1	52.7 ±4.4

Values are mean ± S.D. (n=6); significantly different from

(* control, *p<0.05*),

a (tetracycline , *p*<0.05),

b (alpha, *p*< 0.05),

c (paste base, *p*<0.05),

d (Poly Herbal paste 40%, *p*< 0.05), ( PHP: Poly Herbal Paste ).

*Histopathological study*

Skin histopathology 

**Figure 1:**
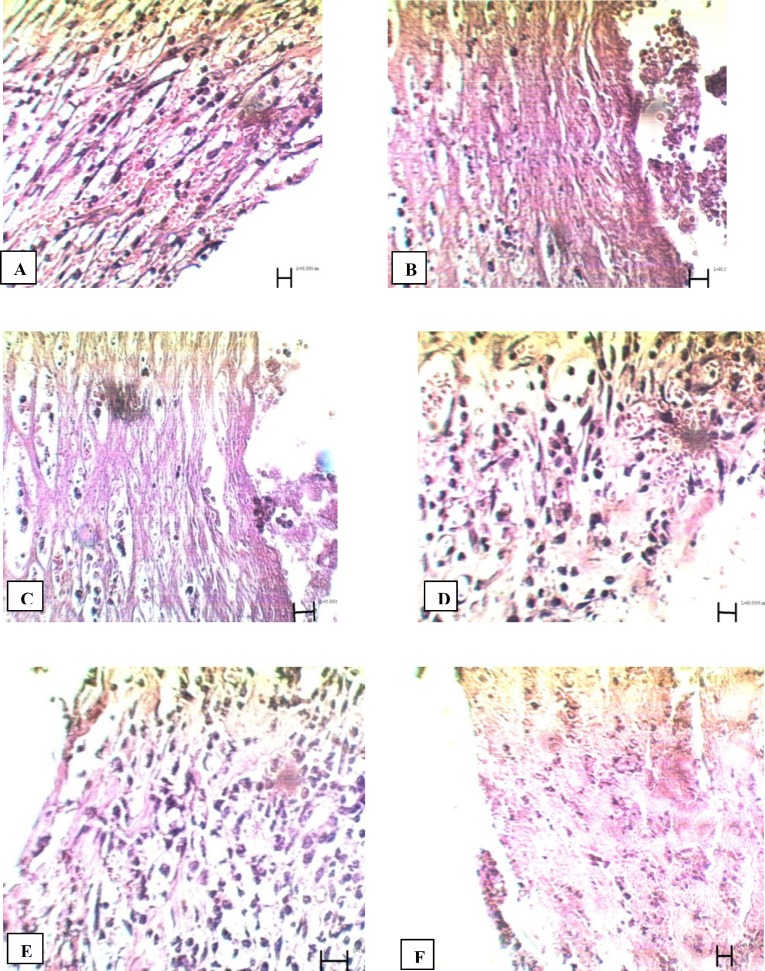
Microscopic panel of wounds on the 14^th ^day of treatment in rats. A) Control skin: The presence of invasive inflammatory cells is evident; no epithelial layer is seen. Vacuolization of the dermal cells, as well as adipose tissue substitution as indexes of immaturity, are evident. b) Tetracycline treated skin: Hemorrhage is relatively high. High density of fibroblasts and low density of blood capillaries are present around the wound. C) Alpha treated skin: Large numbers of blood capillaries are present in and around the wound. In some areas of the wound margins re-epithelialization can be seen. D) PHP 40% treated skin: Many blood capillaries exist in the wound; high density of fibroblasts is seen. E) PHP 10% treated skin: Re-epithelialization, high density of fibroblasts and blood capillaries are seen in the wound. F) Paste base treated skin: Fibroblasts are seen, but are less compared to the treated group; high density of inflammatory cells are present. PHP: poly herbal paste. ×400

**Figure 2:**
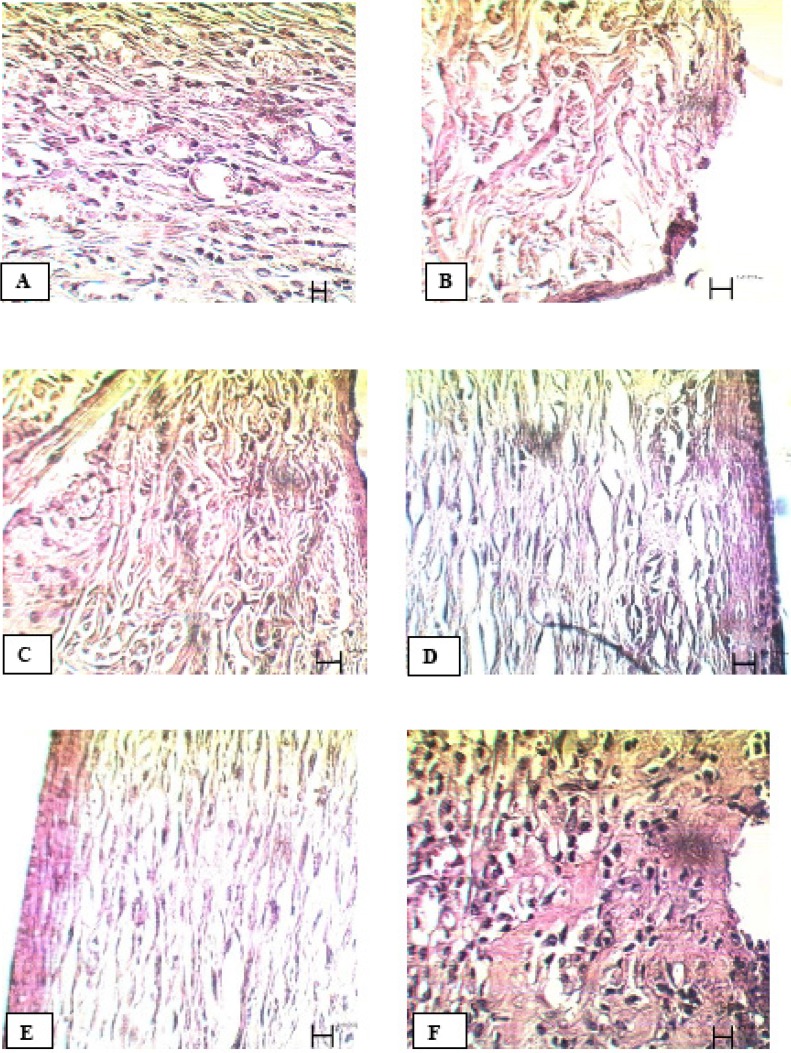
Microscopic panel of wounds on the 21^st ^day of treatment in rats. A) Control skin: new epithelial layer is forming. There is a reduction in wound size. Congestion of inflammatory cells is reduced. B) Tetracycline treated skin: Epithelial is formed but epithelial layers are not yet complete. A large number of fibroblasts and abnormal density of collagen fibers in the dermis are evident. C) Alpha treated skin: Epithelial layer is formed but it is not yet complete. Collagen fibers, blood capillaries, hair follicles and sebaceous glands are evident in the dermis with normal density. D) PHP 40% treated skin: the epidermis, except the horny layer, is formed. Epidermis, hair follicles and sebaceous glands are observed as normal. A normal density of collagen fibers and a normal distribution of connective tissue in the dermis are seen. E) PHP 10% treated skin: Complete formation of the epidermis, except horny layer; high density of blood capillaries in the dermis is seen. In view of the dermis, collagen fibers and connective tissue are completely normal. F) Paste base treated skin: Epithelial layer is formed, but not completely. Low density of fibroblasts and low density of blood capillaries are seen. PHP; poly herbal paste. ×400

Comparison of tissue section from PHP 10%-treated rats with control group showed significant improvement in wound healing in PHP 10%-treated group. The microscopic views of all groups on the14^th ^and 21^st^ days are shown in Figures 1 and 2. 

14^th^ day: In control group, high density of inflammatory cells and excessive bleeding were seen at the site of the lesion; no epithelial layer was formed (figure1A). In tetracycline group, hemorrhage was relatively high. High density of inflammatory cells and low density of blood capillaries were present around the wound (figure 1B). In Alpha group, large numbers of blood capillaries could be seen in and around the wound. Re-epithelialization was seen in some areas of the wound margins (figure 1C). In PHP 40% group, many blood capillaries were seen in the wound, along with high density of fibroblasts (figure 1D). In PHP10% group, re-epithelialization and a high density of fibroblasts and blood capillaries were observed in the wound (figure1E). In the paste base group, fibroblasts were present, but were less in comparison to the treated group. Also, inflammatory cells were present. Blood capillaries were much less than PHP 10% and PHP 40% groups (figure 1F).

21^st^ day: In the control group, new epithelial layer was forming. Wound size reduced. A reduction in congestion of inflammatory cells and blood capillaries were seen (figure 2A). In tetracycline group, epithelial layer was formed but not completely. A large number of fibroblasts and abnormal density of collagen fibers were present in the dermis (figure 2B). In Alpha group, epithelial layer was formed but not completely. Normal density of collagen fibers, blood capillaries, hair follicles and sebaceous glands were observed in the dermis (figure2C). In PHP 40% group, the epidermis, except horny layer, was formed. Epidermis, hair follicles and sebaceous glands were observed as normal. Normal density of collagen fibers and normal distribution of connective tissue in the dermis were seen (figure 2D). In PHP 10% group, complete formation of the epidermis, except horny layer, and high density of blood capillaries in the dermis were evident. In view of the dermis, collagen fibers and connective tissue were completely normal (figure 2E). In paste base group, epithelial layer was formed but not completely. Low density of fibroblasts and blood capillaries were seen (figure 2)

*Kidney histopathology*

**Figure 3:**
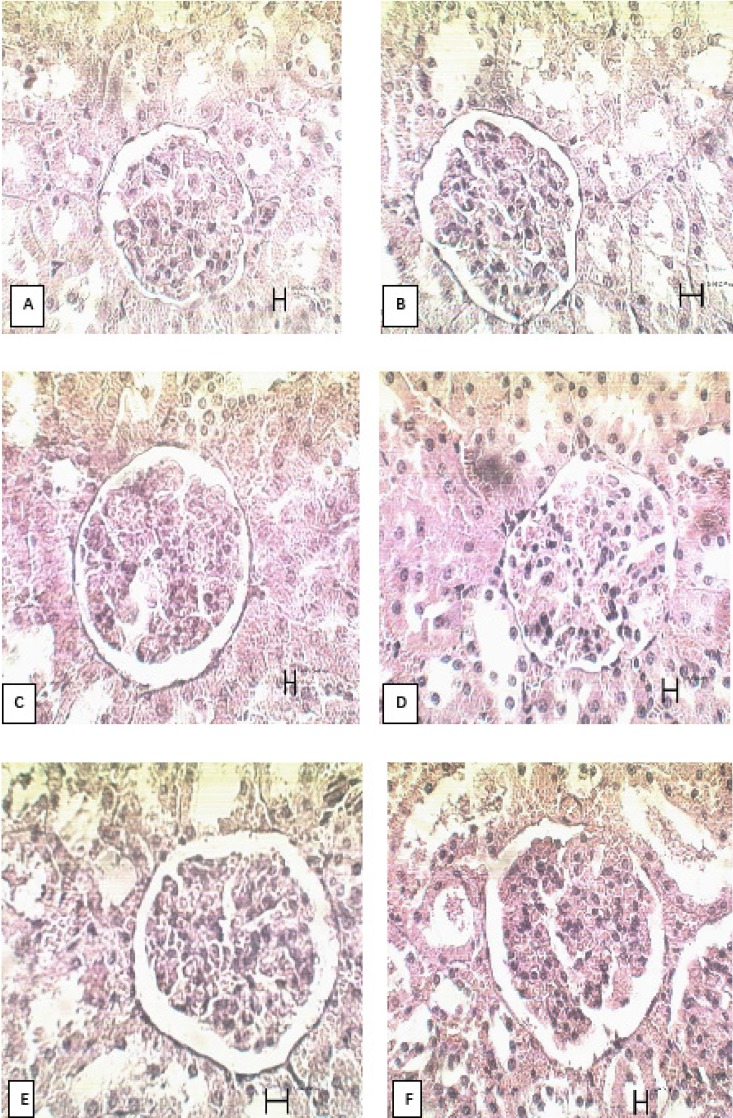
Microscopic views of kidney glomeruli on the 21^st ^day of treatment in rats. A) Control group B) tetracycline group C) Alpha group D) PHP 10% E) PHP 40% F) paste base. ×400

 There was no significant difference in glomerular size of kidney between the PHP and the other groups**. **The results are presented in table 2 and the microscopic views on the 21^st^ day are shown in Figure 3.

**Table 2:** Glomerular size of kidney

**Glomerular size of kidney**		
**Group **	14^th^ day	21^st^ day
control	766.2 ±49.4	673.6 ±6.8
tetracycline	787.9 ±39.0	739.0 ±20.5
alpha	828.5 ±16.0	755.4 ±32.6
PHP 40%	730.3 ±22.0	736.0 ±38.9
PHP 10%	738.8 ±14.8	790.8 ±23.8
paste base	750.1 ±21.0	761.1 ±26.1

Values are mean ± S.D. (n=6); PHP: Poly Herbal Paste.

*DPPH free radical scavenging activity*

Methanol fraction of PHP exhibited dose dependent inhibition of the DPPH. IC_50_ value for PHP methanol fraction was 7.14 mg/mL.

## Discussion

The aim of wound care is lowering the incidence of risk factors that inhibit wound healing, enhancing the healing process and reducing the incidence of wound infections. Many medicinal plants have been found useful in wound healing (3, 29). In the present investigation, wound healing activity of a paste formulation retrieved from ITM sources containing different concentrations of* A. vera*, *C. myrrha* and *B. carteri* has been studied. The results showed that PHP 10% had the best wound healing effects on the 7^th ^day of the treatment, but there was no difference between PHP10%, 40% and Alpha ointment as positive control on the 14^th^ and 21^st ^days. Meanwhile, histopathological study revealed that re-epithelialization was significantly better in PHP10% treated group than other groups. Also, density of blood capillaries (an effective factor in wound healing) in the wound area of PHP 10% treated group was very high and significantly more than other groups. Moreover, there was no renal toxicity in PHP treated rats. Therefore, PHP 10% could accelerate wound healing process compared to other groups.

For a plant to be an efficient wound healer, its active constituents need to have anti-inflammatory, antimicrobial and antioxidant activities. These are the key biological activities that are paid attention to for development of new products for wound healing (25). Previous studies have shown that terpenoids (the main constituents of *C. myrrha*), especially sesqui- and triterpenoids, have anti-inflammatory, analgesic, antiparasitic, antimicrobial, antioxidant and antiulcer activities (16). It has been demonstrated that the methanol extract of *Boswellia carteri* has anti-inflammatory activity (10). Moreover, the antimicrobial activity of *Boswellia spp.* has been observed (12, 30). *Aloe vera,* known as “the healing plant”, has been demonstrated to be effective during healing process in various tissues. The plant plays its healing role through fibroblast proliferation, angiogenesis, production of different growth factors, synthesis of extra-cellular matrix components such as hyaluronic acid, dermatan sulfate and collagen, as well as increasing the amount of cross-links between the collagen molecules in skin, bone fractures and gastric lesion (31). Previous studies have shown that *A. vera* gel is efficient in treatment of inflammation and wound and burn healing. It has also been reported that mannose-6-phosphate, an active constituent in *A. vera*, has wound healing and anti-inflammation activity. In addition, the antioxidant effect of acemannan, another component of *A. vera*, has been revealed (32, 33). Therefore, the paste containing *A. vera*, *C. myrrha* and *B. carteri* could be considered a suitable preparation in wound healing with regard to its ingredients.

## Conclusion

The present study demonstrated that a PHP consisting of 10% herbal mixture of *A. vera*, *C. myrrha* and *B. carteri* has potent wound healing activity in rats. The healing properties of the PHP might be due to several mechanisms, including increasing the rate of re-epithelialization and neovascularization, scavenging of destructive free radicals, reduction of inflammation and control of infection through the antioxidant, anti-inflammatory and antimicrobial effects of the phytoconstituents of the paste. Thus, the present research complemented the use of *Aloe vera*, *Boswellia carteri* and *Commiphora myrrha *in treatment of wounds as registered in prescriptions in ITM sources. Hence, this PHP could be considered as a potential topical product for wound healing. However, further clinical experiments are necessary.
